# Supplementary material for: A rapid and accurate method for the detection of four aminoglycoside modifying enzyme drug resistance gene in clinical strains of Escherichia coli by a multiplex polymerase chain reaction
Source: PeerJ. 2020 Apr 10;8:e8944. doi: 10.7717/peerj.8944 (PMC7153551; doi:10.7717/peerj.8944)
Supplement: Supplemental Information 1 — Note: SPEC_NUM and SPEC_TYPE means the specimen number and type. AMK_NM, GEN_NM, and TOB_NM means the resistance of amikacin, gentamicin, and tobramycin antibiotics. SP, UR, SE, BF,DG, BL, SW, ST means the sputum, clearing urine, secretion, excreta, blood, surgical incision, and stool of the patients, respectively. [file peerj-08-8944-s001.doc]

**Supplementary materials**

**Table S1** **Resistance genes information and the detection results of S-PCR, M-PCR of 237 clinical *Escherichia coli***

|  | Resistance genes | | | | Simplex PCR | | | | Multiplex PCR | | | |
| --- | --- | --- | --- | --- | --- | --- | --- | --- | --- | --- | --- | --- |
| SPEC_NUM | *Aac(6’)-**Ib* | *Ant(3’’)**-Ia* | *Aph(3’)-Ia* | *Aac(3)-II* | *Aac(6’)-Ib* | *Ant(3’’)-Ia* | *Aph(3’)-Ia* | *Aac(3)-II* | *Aac(6’)-Ib* | *Ant(3’’)-Ia* | *Aph(3’)-Ia* | *Aac(3)-II* |
| 1607UR0030 | + | - | - | + | + | - | - | + | + | - | - | + |
| 1607UR0095 | + | + | - | + | + | + | - | + | + | + | - | + |
| 1611SE0056 | + | + | + | + | + | + | + | + | + | + | + | + |
| 1611970016 | + | - | - | - | + | - | - | - | + | - | - | - |
| 1611NY0004 | + | + | + | + | + | + | + | + | + | + | + | + |
| 1611BL0104 | + | - | - | + | + | - | - | + | + | - | - | + |
| 1611UR0083 | + | - | + | + | + | - | + | + | + | - | + | + |
| 1611UR0279 | + | - | - | + | + | - | - | + | + | - | - | + |
| 1611SE0050 | + | - | - | - | + | - | - | - | + | - | - | - |
| 1606BL0196 | + | - | - | + | + | - | - | + | + | - | - | + |
| 1611BL1143 | + | + | + | + | + | + | + | + | + | + | + | + |
| 1611UR0140 | + | - | - | + | + | - | - | + | + | - | - | + |
| 1611UR0148 | + | - | - | - | + | - | - | - | + | - | - | - |
| 1611970084 | + | - | - | + | + | - | - | + | + | - | - | + |
| 1611UR0282 | + | + | + | + | + | + | + | + | + | + | + | + |
| 1611SE0187 | + | - | - | + | + | - | - | + | + | - | - | + |
| 1611UR0205 | + | - | - | + | + | - | - | + | + | - | - | + |
| 1611SP0549 | + | + | + | + | + | + | + | + | + | + | + | + |
| 1611SP0865 | + | + | + | + | + | + | + | + | + | + | + | + |
| 1611UR0345 | + | + | + | + | + | + | + | + | + | + | + | + |
| 1611SE0236 | + | - | - | + | + | - | - | + | + | - | - | + |
| 1611SE0260 | + | - | - | + | + | - | - | + | + | - | - | + |
| 1611SE0252 | + | - | - | + | + | - | - | + | + | - | - | + |
| 1611UR0062 | + | + | + | + | + | + | + | + | + | + | + | + |
| 1611UR0045 | + | + | - | + | + | + | - | + | + | + | - | + |
| 1611970115 | + | + | + | + | + | + | + | + | + | + | + | + |
| 1611UR0215 | + | + | + | + | + | + | + | + | + | + | + | + |
| 1611SE0096 | + | + | - | + | + | + | - | + | + | + | - | + |
| 1611SE0206 | + | - | + | + | + | - | + | + | + | - | + | + |
| 1611UR0046 | + | + | + | + | + | + | + | + | + | + | + | + |
| 1611SE0166 | + | - | + | + | + | - | + | + | + | - | + | + |
| 1611970100 | + | + | + | + | + | + | + | + | + | + | + | + |
| 1611SE0258 | + | - | - | - | + | - | - | - | + | - | - | - |
| 1611UR0318 | + | + | + | + | + | + | + | + | + | + | + | + |
| 1611UR0314 | + | - | - | + | + | - | - | + | + | - | - | + |
| 1611SE0119 | + | - | - | - | + | - | - | - | + | - | - | - |
| 1611SP0361 | + | - | + | + | + | - | + | + | + | - | + | + |
| 1611UR0095 | + | + | + | + | + | + | + | + | + | + | + | + |
| 1607SE0068 | + | - | - | + | + | - | - | + | + | - | - | + |
| 1607UR0248 | + | - | - | - | + | - | - | - | + | - | - | - |
| 1607SP0464 | + | - | - | + | + | - | - | + | + | - | - | + |
| 1607BL0700 | + | + | - | + | + | + | - | + | + | + | - | + |
| 1607UR0170 | + | - | - | - | + | - | - | - | + | - | - | - |
| 1607UR0162 | + | + | - | + | + | + | - | + | + | + | - | + |
| 1607SP0685 | + | + | - | + | + | + | - | + | + | + | - | + |
| 1607UR0081 | + | + | - | - | + | + | - | - | + | + | - | - |
| 1607UR0256 | + | - | - | + | + | - | - | + | + | - | - | + |
| 1607UR0136 | + | - | - | - | + | - | - | - | + | - | - | - |
| 1607UR0130 | + | - | - | + | + | - | - | + | + | - | - | + |
| 1607SE0069 | + | - | - | + | + | - | - | + | + | - | - | + |
| 1607SP0675 | + | - | - | + | + | - | - | + | + | - | - | + |
| 1607SE0147 | + | - | - | + | + | - | - | + | + | - | - | + |
| 1607SP0720 | + | - | - | - | + | - | - | - | + | - | - | - |
| 1607BL0805 | + | - | - | - | + | - | - | - | + | - | - | - |
| 1606SE0082 | + | - | - | - | + | - | - | - | + | - | - | - |
| 1607UR0210 | + | - | - | + | + | - | - | + | + | - | - | + |
| 1607UR0253 | + | - | + | + | + | - | + | + | + | - | + | + |
| 1607SE0133 | + | - | - | + | + | - | - | + | + | - | - | + |
| 1607SE0029 | + | + | - | + | + | + | - | + | + | + | - | + |
| 1606UR0356 | + | + | - | + | + | + | - | + | + | + | - | + |
| 1607SE0034 | + | + | - | - | + | + | - | - | + | + | - | - |
| 1607NY0002 | + | - | - | + | + | - | - | + | + | - | - | + |
| 1607UR0012 | + | - | - | + | + | - | - | + | + | - | - | + |
| 1607BL0146 | + | - | - | - | + | - | - | - | + | - | - | - |
| 1606SP0953 | + | - | - | + | + | - | - | + | + | - | - | + |
| 1606BF0192 | + | - | - | + | + | - | - | + | + | - | - | + |
| 1606UR0343 | + | - | - | + | + | - | - | + | + | - | - | + |
| 1607NY0001 | + | - | - | - | + | - | - | - | + | - | - | - |
| 1606UR0337 | + | - | - | + | + | - | - | + | + | - | - | + |
| 1607UR0062 | + | - | - | - | + | - | - | - | + | - | - | - |
| 1606SP0795 | + | - | - | - | + | - | - | - | + | - | - | - |
| 1606SE0143 | + | - | - | - | + | - | - | - | + | - | - | - |
| 1606UR0315 | + | - | - | - | + | - | - | - | + | - | - | - |
| 1606SW0015 | + | - | - | - | + | - | - | - | + | - | - | - |
| 1606UR0275 | + | - | - | + | + | - | - | + | + | - | - | + |
| 1606BL1523 | + | + | - | + | + | + | - | + | + | + | - | + |
| 1606BF0134 | + | + | - | - | + | + | - | - | + | + | - | - |
| 1606SE0213 | + | - | - | + | + | - | - | + | + | - | - | + |
| 1607SE0075 | - | - | - | + | - | - | - | + | - | - | - | + |
| 1607BL0339 | - | - | - | + | - | - | - | + | - | - | - | + |
| 1611UR0123 | - | - | + | + | - | - | + | + | - | - | + | + |
| 1611UR0290 | - | - | - | + | - | - | - | + | - | - | - | + |
| 1611SE0250 | - | + | + | + | - | + | + | + | - | + | + | + |
| 1611UR0338 | - | + | - | + | - | + | - | + | - | + | - | + |
| 1611UR0125 | - | - | - | + | - | - | - | + | - | - | - | + |
| 1611BL0313 | - | - | - | + | - | - | - | + | - | - | - | + |
| 1611SE0078 | - | + | + | + | - | + | + | + | - | + | + | + |
| 1611970086 | - | - | - | + | - | - | - | + | - | - | - | + |
| 1611BL1029 | - | + | + | + | - | + | + | + | - | + | + | + |
| 1611SE0090 | - | - | + | + | - | - | + | + | - | - | + | + |
| 1611SP0934 | - | - | + | + | - | - | + | + | - | - | + | + |
| 1611UR0351 | - | - | - | + | - | - | - | + | - | - | - | + |
| 1611SE0259 | - | - | - | + | - | - | - | + | - | - | - | + |
| 1611SE0105 | - | - | + | + | - | - | + | + | - | - | + | + |
| 1611UR0305 | - | - | + | + | - | - | + | + | - | - | + | + |
| 1611UR0119 | - | - | - | + | - | - | - | + | - | - | - | + |
| 1611BL0384 | - | - | - | + | - | - | - | + | - | - | - | + |
| 1611NY0016 | - | - | - | + | - | - | - | + | - | - | - | + |
| 1611UR0287 | - | - | - | + | - | - | - | + | - | - | - | + |
| 1611SE0138 | - | + | + | + | - | + | + | + | - | + | + | + |
| 1611UR0105 | - | - | - | + | - | - | - | + | - | - | - | + |
| 1611BL0397 | - | - | - | + | - | - | - | + | - | - | - | + |
| 1611BF0038 | - | + | + | + | - | + | + | + | - | + | + | + |
| 1607BL1414 | - | - | - | + | - | - | - | + | - | - | - | + |
| 1607UR0221 | - | + | + | + | - | + | + | + | - | + | + | + |
| 1607UR0247 | - | - | + | + | - | - | + | + | - | - | + | + |
| 1607SE0142 | - | + | - | + | - | + | - | + | - | + | - | + |
| 1607SP0521 | - | - | - | + | - | - | - | + | - | - | - | + |
| 1607UR0150 | - | + | - | + | - | + | - | + | - | + | - | + |
| 1607SE0163 | - | + | - | + | - | + | - | + | - | + | - | + |
| 1607BL0798 | - | + | - | + | - | + | - | + | - | + | - | + |
| 1607UR0216 | - | - | - | + | - | - | - | + | - | - | - | + |
| 1607SE0139 | - | - | - | + | - | - | - | + | - | - | - | + |
| 1607SE0140 | - | - | - | + | - | - | - | + | - | - | - | + |
| 1607SE0088 | - | - | - | + | - | - | - | + | - | - | - | + |
| 1607UR0257 | - | - | - | + | - | - | - | + | - | - | - | + |
| 1607UR0166 | - | - | - | + | - | - | - | + | - | - | - | + |
| 1607SE0114 | - | - | - | + | - | - | - | + | - | - | - | + |
| 1607UR0124 | - | + | - | + | - | + | - | + | - | + | - | + |
| 1611SP0074 | - | - | - | + | - | - | - | + | - | - | - | + |
| 1607UR0107 | - | - | - | + | - | - | - | + | - | - | - | + |
| 1607UR0167 | - | + | - | + | - | + | - | + | - | + | - | + |
| 1607SE0096 | - | + | + | + | - | + | + | + | - | + | + | + |
| 1607SE0098 | - | - | - | + | - | - | - | + | - | - | - | + |
| 1607BL1256 | - | - | - | + | - | - | - | + | - | - | - | + |
| 1607BF0122 | - | - | - | + | - | - | - | + | - | - | - | + |
| 1607BL0394 | - | - | - | + | - | - | - | + | - | - | - | + |
| 1611UR0030 | - | + | - | + | - | + | - | + | - | + | - | + |
| 1607BF0123 | - | - | - | + | - | - | - | + | - | - | - | + |
| 1607BF0060 | - | - | - | + | - | - | - | + | - | - | - | + |
| 1607UR0143 | - | - | - | + | - | - | - | + | - | - | - | + |
| 1607UR0191 | - | + | - | + | - | + | - | + | - | + | - | + |
| 1607SE0120 | - | - | - | + | - | - | - | + | - | - | - | + |
| 1607BF0076 | - | + | + | + | - | + | + | + | - | + | + | + |
| 1611SP0124 | - | - | - | + | - | - | - | + | - | - | - | + |
| 1607SP0436 | - | - | - | + | - | - | - | + | - | - | - | + |
| 1611UR0023 | - | - | - | + | - | - | - | + | - | - | - | + |
| 1607UR0133 | - | + | - | + | - | + | - | + | - | + | - | + |
| 1607SE0104 | - | - | - | + | - | - | - | + | - | - | - | + |
| 1607SP0030 | - | - | - | + | - | - | - | + | - | - | - | + |
| 1607UR0053 | - | - | + | + | - | - | + | + | - | - | + | + |
| 1607SE0014 | - | - | - | + | - | - | - | + | - | - | - | + |
| 1607SP0048 | - | - | - | + | - | - | - | + | - | - | - | + |
| 1606UR0370 | - | - | - | + | - | - | - | + | - | - | - | + |
| 1607UR0008 | - | - | - | + | - | - | - | + | - | - | - | + |
| 1607SE0009 | - | - | - | + | - | - | - | + | - | - | - | + |
| 1606SE0201 | - | - | - | + | - | - | - | + | - | - | - | + |
| 1606BL1640 | - | - | - | + | - | - | - | + | - | - | - | + |
| 1607SE0041 | - | + | - | + | - | + | - | + | - | + | - | + |
| 1607BF0009 | - | - | - | + | - | - | - | + | - | - | - | + |
| 1606UR0339 | - | - | - | + | - | - | - | + | - | - | - | + |
| 1606BL1768 | - | - | + | + | - | - | + | + | - | - | + | + |
| 1607SE0045 | - | + | - | + | - | + | - | + | - | + | - | + |
| 1606UR0337 | - | - | - | + | - | - | - | + | - | - | - | + |
| 1607SE0044 | - | + | - | + | - | + | - | + | - | + | - | + |
| 1607UR0009 | - | - | + | + | - | - | + | + | - | - | + | + |
| 1607SE0051 | - | - | - | + | - | - | - | + | - | - | - | + |
| 1606UR0292 | - | - | - | + | - | - | - | + | - | - | - | + |
| 1606UR0310 | - | - | - | + | - | - | - | + | - | - | - | + |
| 1606UR0267 | - | + | - | + | - | + | - | + | - | + | - | + |
| 1606UR0268 | - | + | + | + | - | + | + | + | - | + | + | + |
| 1607SE0042 | - | - | - | + | - | - | - | + | - | - | - | + |
| 1611UR0109 | - | + | - | - | - | + | - | - | - | + | - | - |
| 1607UR0004 | - | + | - | - | - | + | - | - | - | + | - | - |
| 1607SE0048 | - | + | - | - | - | + | - | - | - | + | - | - |
| 1607UR0085 | - | + | - | - | - | + | - | - | - | + | - | - |
| 1611BL1405 | - | + | - | - | - | + | - | - | - | + | - | - |
| 1607UR0043 | - | - | + | - | - | - | + | - | - | - | + | - |
| 1611UR0339 | - | - | - | - | - | - | - | - | - | - | - | - |
| 1611UR0274 | - | - | - | - | - | - | - | - | - | - | - | - |
| 1611UR0102 | - | - | - | - | - | - | - | - | - | - | - | - |
| 1611UR0084 | - | - | - | - | - | - | - | - | - | - | - | - |
| 1611UR0074 | - | - | - | - | - | - | - | - | - | - | - | - |
| 1611UR0073 | - | - | - | - | - | - | - | - | - | - | - | - |
| 1611UR0050 | - | - | - | - | - | - | - | - | - | - | - | - |
| 1611UR0041 | - | - | - | - | - | - | - | - | - | - | - | - |
| 1611UR0027 | - | - | - | - | - | - | - | - | - | - | - | - |
| 1611SP0888 | - | - | - | - | - | - | - | - | - | - | - | - |
| 1611SP0260 | - | - | - | - | - | - | - | - | - | - | - | - |
| 1611SP0182 | - | - | - | - | - | - | - | - | - | - | - | - |
| 1611SP0046 | - | - | - | - | - | - | - | - | - | - | - | - |
| 1611SE0253 | - | - | + | - | - | - | + | - | - | - | + | - |
| 1611SE0208 | - | - | - | - | - | - | - | - | - | - | - | - |
| 1611SE0200 | - | - | - | - | - | - | - | - | - | - | - | - |
| 1611SE0057 | - | - | - | - | - | - | - | - | - | - | - | - |
| 1611SE0054 | - | - | - | - | - | - | - | - | - | - | - | - |
| 1611SE0032 | - | - | - | - | - | - | - | - | - | - | - | - |
| 1611NY0006 | - | - | - | - | - | - | - | - | - | - | - | - |
| 1611BL0614 | - | - | - | - | - | - | - | - | - | - | - | - |
| 1611BL0261 | - | - | - | - | - | - | - | - | - | - | - | - |
| 1611970115 | - | - | + | - | - | - | + | - | - | - | + | - |
| 1611970100 | - | + | - | - | - | + | - | - | - | + | - | - |
| 1611970084 | - | - | - | - | - | - | - | - | - | - | - | - |
| 1611970016 | - | - | - | - | - | - | - | - | - | - | - | - |
| 1607UR0230 | - | - | - | - | - | - | - | - | - | - | - | - |
| 1607UR0154 | - | - | - | - | - | - | - | - | - | - | - | - |
| 1607UR0094 | - | + | - | - | - | + | - | - | - | + | - | - |
| 1607UR0058 | - | - | - | - | - | - | - | - | - | - | - | - |
| 1607UR0030 | - | - | - | - | - | - | - | - | - | - | - | - |
| 1607UR0026 | - | - | - | - | - | - | - | - | - | - | - | - |
| 1607SP0543 | - | - | - | - | - | - | - | - | - | - | - | - |
| 1607SP0150 | - | - | - | - | - | - | - | - | - | - | - | - |
| 1607SE0178 | - | - | - | - | - | - | - | - | - | - | - | - |
| 1607SE0149 | - | - | - | - | - | - | - | - | - | - | - | - |
| 1607SE0083 | - | - | - | - | - | - | - | - | - | - | - | - |
| 1607SE0075 | - | - | - | - | - | - | - | - | - | - | - | - |
| 1607SE0070 | - | - | - | - | - | - | - | - | - | - | - | - |
| 1607SE0052 | - | - | - | - | - | - | - | - | - | - | - | - |
| 1607SE0010 | - | - | - | - | - | - | - | - | - | - | - | - |
| 1607NY0010 | - | - | - | - | - | - | - | - | - | - | - | - |
| 1607BL1430 | - | - | - | - | - | - | - | - | - | - | - | - |
| 1607BL1242 | - | - | - | - | - | - | - | - | - | - | - | - |
| 1607BL1160 | - | - | - | - | - | - | - | - | - | - | - | - |
| 1607BL0911 | - | - | - | - | - | - | - | - | - | - | - | - |
| 1607BL0729 | - | - | - | - | - | - | - | - | - | - | - | - |
| 1607BL0395 | - | - | - | - | - | - | - | - | - | - | - | - |
| 1607BL0339 | - | - | - | - | - | - | - | - | - | - | - | - |
| 1607BL0316 | - | - | - | - | - | - | - | - | - | - | - | - |
| 1607BL0229 | - | - | - | - | - | - | - | - | - | - | - | - |
| 1606UR0367 | - | - | - | - | - | - | - | - | - | - | - | - |
| 1606UR0360 | - | - | - | - | - | - | - | - | - | - | - | - |
| 1606UR0335 | - | - | - | - | - | - | - | - | - | - | - | - |
| 1606UR0328 | - | - | - | - | - | - | - | - | - | - | - | - |
| 1606UR0270 | - | - | - | - | - | - | - | - | - | - | - | - |
| 1606UR0269 | - | - | - | - | - | - | - | - | - | - | - | - |
| 1606SP0738 | - | - | - | - | - | - | - | - | - | - | - | - |
| 1606SP0614 | - | - | - | - | - | - | - | - | - | - | - | - |
| 1606SE0199 | - | - | - | - | - | - | - | - | - | - | - | - |
| 1606SE0192 | - | - | - | - | - | - | - | - | - | - | - | - |
| 1606NY0026 | - | - | - | - | - | - | - | - | - | - | - | - |
| 1606BL1638 | - | - | - | - | - | - | - | - | - | - | - | - |
| 1606BL1618 | - | - | - | - | - | - | - | - | - | - | - | - |
| 1606BF0171 | - | - | - | - | - | - | - | - | - | - | - | - |
| 1611970115 | - | - | - | - | - | - | - | - | - | - | - | - |
| 1611970100 | - | - | - | - | - | - | - | - | - | - | - | - |
| 1611970084 | - | - | - | - | - | - | - | - | - | - | - | - |
| 1611970016 | - | - | - | - | - | - | - | - | - | - | - | - |

**Note:** SPEC_NUM and SPEC_TYPE means the specimen number and type. AMK_NM, GEN_NM, and TOB_NM means the resistance of amikacin, gentamicin, and tobramycin antibiotics. SP, UR, SE, BF,DG, BL, SW, ST means the [sputum](http://dict.cnki.net/dict_result.aspx?searchword=痰液&tjType=sentence&style=&t=sputum), [clearing urine](http://dict.cnki.net/dict_result.aspx?searchword=清洁中段尿&tjType=sentence&style=&t=clearing+urine), [secretion](http://dict.cnki.net/dict_result.aspx?searchword=分泌物&tjType=sentence&style=&t=secretion), [excreta](http://dict.cnki.net/dict_result.aspx?searchword=排泄物&tjType=sentence&style=&t=excreta), blood, [surgical incision](http://dict.cnki.net/dict_result.aspx?searchword=手术切口&tjType=sentence&style=&t=surgical+incision), and stool of the patients, respectively.
