# Supplementary figures and images for: A rapid and accurate method for the detection of four aminoglycoside modifying enzyme drug resistance gene in clinical strains of Escherichia coli by a multiplex polymerase chain reaction
Source: PeerJ. 2020 Apr 10;8:e8944. doi: 10.7717/peerj.8944 (PMC7153551; doi:10.7717/peerj.8944)

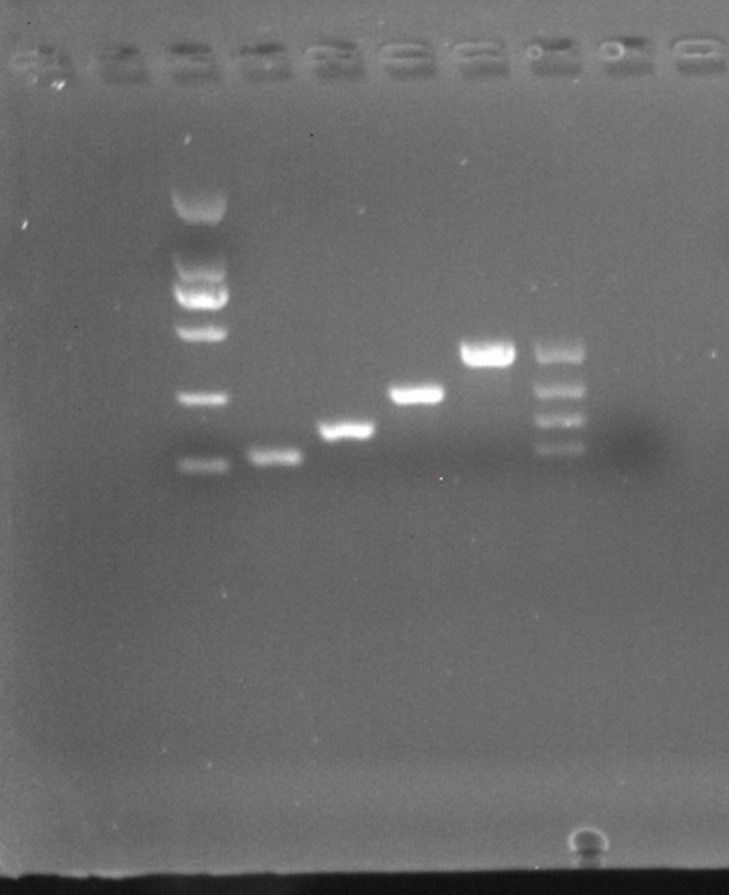

Supplement: Supplemental Information 2 [file peerj-08-8944-s002.png]

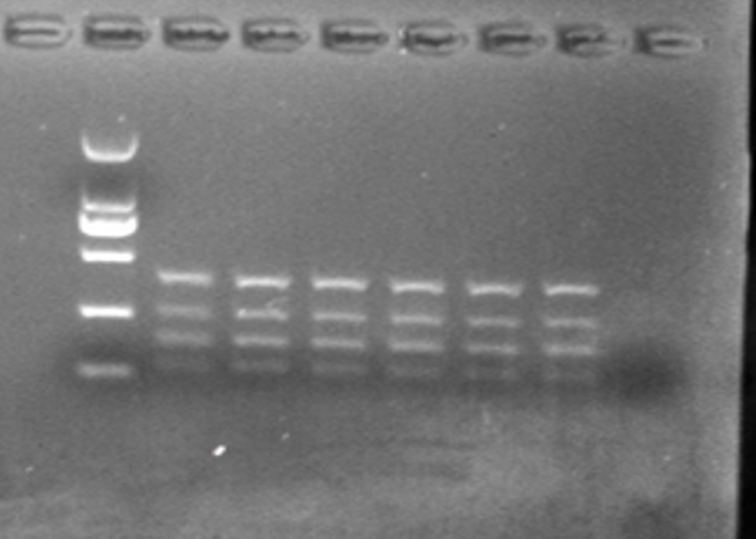

Supplement: Supplemental Information 3 [file peerj-08-8944-s003.png]

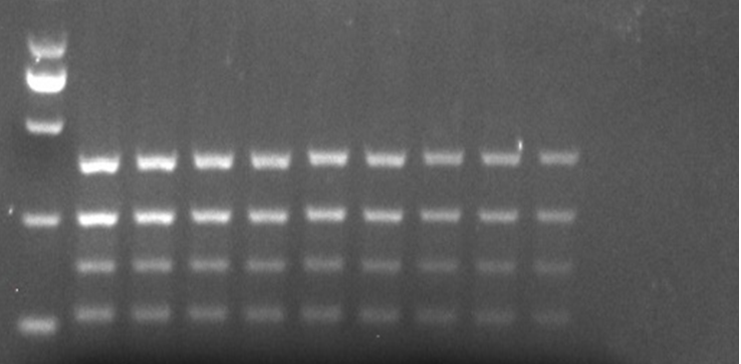

Supplement: Supplemental Information 4 [file peerj-08-8944-s004.png]

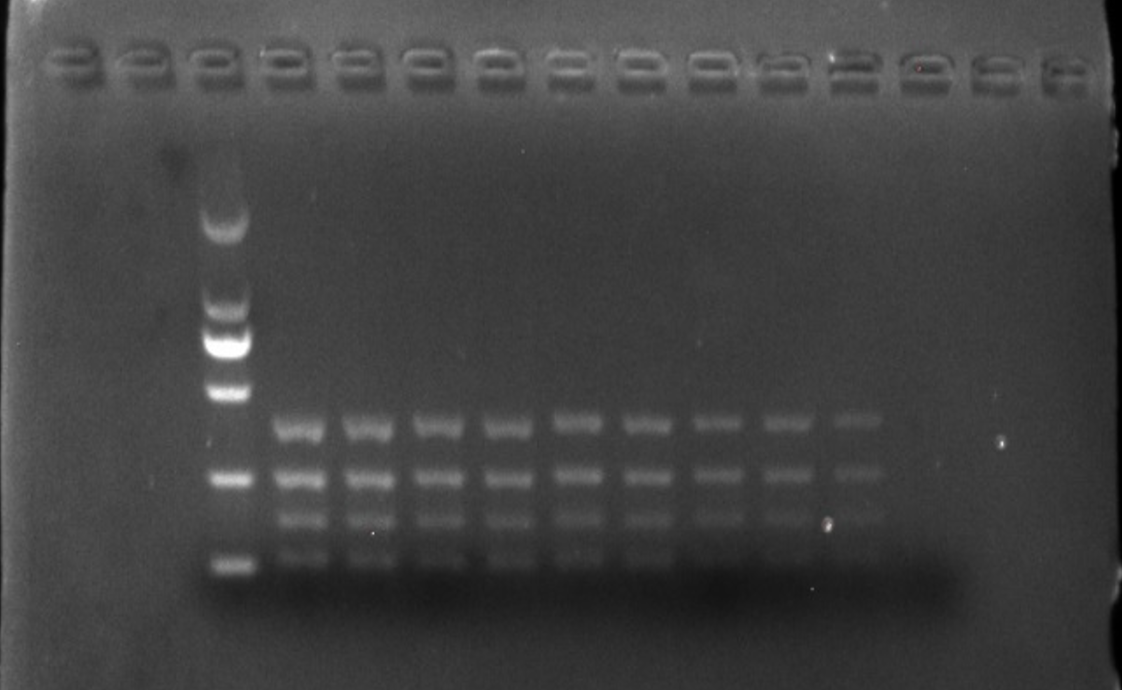

Supplement: Supplemental Information 5 [file peerj-08-8944-s005.png]
